# Visual biases from cis and trans judges in evaluation of voices with pitch and timbre manipulations

**DOI:** 10.3389/fpsyg.2026.1755303

**Published:** 2026-09-01

**Authors:** Jay Marchand Knight, Mickael L. D. Deroche

**Affiliations:** Laboratory for Hearing and Cognition, Department of Psychology, Concordia University, Montreal, QC, Canada

**Keywords:** audio-visual integration, FACH, gender studies, implicit bias, McGurk, pitch, song, speech

## Abstract

When judging the voice of a person, one tends to integrate how this person looks into their voice qualities. This phenomenon is not only misleading but also unfair to many individuals who do not fit voice-body expectations, particularly those who are transgender. Interestingly, trans individuals (as judges) appear less prone to this visual bias, but it is unclear how they remain so. They might conceivably rely on certain characteristics of the voice—typically its pitch or its timbre—to operate in a more unimodal manner, effectively weighing down the influence of visual cues. This hypothesis was explored in two online studies, where cisgender and transgender participants were presented with 18 different actors (on a continuum from bass to soprano) singing or speaking short sentences. While the visual content of the stimuli was unchanged, the audio content was manipulated in fundamental frequency (F0) or in vocal tract length (VTL) without inducing any desynchronization between audio and video. Every participant provided voice type ratings for (1) Audio-only (A) stimuli to test the effect of the manipulation, (2) Video-only (V) stimuli to confirm that the manipulation had no role, and (3) combined Audio-Visual (AV) stimuli to see how much visual cues “contaminated” the audio evaluation in each manipulation. The manipulations worked as expected, lowering A-ratings for lower F0 and longer VTL, while having no impact on V-ratings. The AV-ratings were systematically shifted from A-ratings in the direction of V-ratings, but trans participants were less prone to this bias in experiment 2 (with VTL manipulations), not in experiment 1 (with F0 manipulations). We conclude that the trans advantage may partly originate from a better internalization of timbre cues, while pitch cues have more of a universal impact.

## 1 Introduction

Voices are perceived both aurally and visually, and audio-visual (AV) integration is a necessary—yet sometimes unfortunate—aspect of voice appraisal (Marchand Knight et al., 2023). That vision influences hearing has been known for many years and is not restricted to voices. For example, the perception of percussive sounds can cause us to hear a sound as longer, if the performer elongates their stroke, or shorter if the performer uses a short, sharp stroke, even if the audio signal in both visual presentations is exactly the same (Schutz and Manning, 2012). One might naively think that expertise would prevent such visual influences, but it is not the case: when presented with videos of different pianists pretending to play along with the same recorded audio, musically trained listeners believed they were hearing different performances (Behne, 1990). Even more relevant to this paper, a replication study later found that the women pianists were judged as more dramatic and expressive, while the men pianists were judged to be more precise and authentic, although the listeners were once again, hearing the same audio (Behne and Wöllner, 2011). This visual dominance is sometimes exploited to elicit sound production. For example, opera conductor, Alexis Enyart, describes her work as “The communication of sound through gesture.” That is, she is able to convey mood, dynamics, and tempi, to the orchestra and singers by moving her hands, through her facial expressions, and with whole body gestures, thus influencing the sounds the orchestra produces (Marchand Knight, 2026). Furthermore, gestures made by singers can affect how listeners assign nuanced meanings to lyrics and how clearly key changes are perceived by audiences (Thompson et al., 2005).

Our AV expectations of the voice are based on prior knowledge and enculturation. That is, we have been trained, through established cultural practices, to expect certain bodies to produce certain kinds of sound. Our work seeks to understand, question, and disrupt biased expectations, which are particularly harmful to transgender people but, to some extent, cisgender people as well. Issues regarding prescribed societal gender roles and expectations, incongruous internal and perceived/performed identity, and accessibility affect all people in our societies, however these obstacles disproportionately impact marginalized people. This obviously influences the ways in which people communicate and use their voices to interact (Viola and Madureira, 2007; Carson, 1994).

Digging further into aspects of voice that are believed to influence our perception of gender, here we aim to tease apart the relative importance of fundamental frequency (F0, related to voice pitch) and vocal tract length (VTL, related to voice timbre). In order to test this, we manipulated either the pitch or the timbre of speaking and singing actors to test whether these purely acoustic manipulations would influence voice type classification and force a break with AV integration, when compared to our initial study which demonstrated visual biases in unprocessed stimuli (but the same actors as here) by cis listeners, and to a reduced degree by trans listeners (Marchand Knight et al., 2023).

### 1.1 The trans advantage

Nina Sun Eidsheim (2019) says of the voice:

“Voice is not singular; it is collective. Voice is not innate; it is cultural. Voice's source is not the singer; it is the listener.”

While these statements are provocative, Eidsheim echoes Judith Butler's theory of performativity which states that gender, far from being decided at birth, is

“a mix of a cultural norms, historical formations, family influence, psychic realities, desires, and wishes.” (Big Think, 2023, 2.16).

Butler also explains gender performativity as requiring an audience. These principles are at the heart of psychophysics, which seeks to the bridge the gap between the objective world and our unique subjective experience of it. But it goes beyond individual experiences when we share them and agree on terminology. As Karl Popper framed it: there may be three worlds: (1) an objective world, consisting of sound waves and spectra; (2) a perceptual world, existing as pitch, timbre, or mood within each listener; and (3) a collective world, emerging when we communally assign semantics to the sound waves we have individually perceived (Popper, 1968; Karpiński, 1979). Psychoacoustics is generally framed according to the first two, while the third world is much less appreciated. What this means for the transgender voice user, wishing to align their voice with their inner sense of gender and their outward gender presentation, is that they must not only consider their own objective vocal anatomy with their desired perceptual impact but also reconcile these with societal ideas of masculinity and femininity. This places tremendous pressure on the trans voice user and risks limiting timbral imagination and thus possibility for vocal production, as we are molded by a system of rewards and punishment to be more “feminine” or more “masculine” and to believe that there are limitations (which may or may not exist) on our voice's possibility. Trans people challenge us to reconsider the gender norms we have defined through this collective system; however, we are far from a Butlerian utopia in which gender is not enforced. To exist comfortably and safely in real life where gender is still strongly politicized (especially in recent times in North America and elsewhere), many trans people understandably wish to perform their gender as completely as possible. Gender performativity, as first defined by Judith Butler, means behaving in ways that are believed to be recognized as masculine or feminine. This may mean, among other things, wearing certain clothing, walking a certain way, and speaking a certain way (Butler, 2003, 1988). Therefore, we must understand which aspects of voice most influence the listeners' perception of gender. Only then, can the trans voice user make informed decisions about which aspects of their own voice they wish to modify.

In our previous study (Marchand Knight et al., 2023), we discovered that when compared with cisgender participants, transgender participants displayed 30% less visual bias in the evaluation of gendered voice sounds. To clarify, we presented two groups of participants (one trans group and one cis group) with audio alone (A), video alone (V), and AV combinations of different actors (speaking or singing) and asked participants to guess the voice type of the actor on a continuous scale from bass to soprano. We then assessed whether the ratings of the AV versions differed from those of the A versions and found that they were systematically dragged in the direction of the V stimuli. In essence, this demonstrated that judging a voice is not a purely auditory process, which is not so surprising in itself given the multisensory integration capabilities of our brains (McGurk and MacDonald, 1976), and more specifically, that our brains use AV integration regularly when listening, for example for sound localization (ventriloquist effect) (Howard and Templeton, 1966) and to interpret speech in noisy environments (cocktail party problem) (Cherry, 1953). More interestingly, cisgender participants (as judges) were more influenced by the physical appearance of the actor than trans participants (as judges). That is, cis individuals displayed a larger visual bias, based on their preconceived ideas of multisensory gender, and because of this, were more prone to assess the actors' voice as a function of their physical appearance. We surmised that this group difference was due to the lived experiences of trans people and more openness toward subversions of gendered expectations, especially that anatomy should always influence interests and behaviors (Marchand Knight et al., 2023). We refer to this phenomenon here as the “trans advantage.” In this initial study, no manipulation was performed on the audios, the videos, or the integrated AV versions, but the size of the bias varied across the actors (who naturally had different voice pitch and timbre). To further explore the phenomenon, here we sought to isolate pitch and timbre *within the same actors* to assess which cues judges would rely upon most to assess voice type, and by extension, actor gender. To achieve this, we performed manipulations on the audio only and reintegrated the processed stimuli seamlessly into the video of the original speakers (i.e., there was never any AV combination taken from different A and V materials).

### 1.2 Strength of pitch and timbre qualities in voice

Gender-affirming voice specialists, that is voice researchers and teachers as well as speech language pathologists who work primarily with gender expansive clients, have identified several factors that influence gender perception in the voice. These include pitch, timbre, melodic contour, mode of emphasis (auditory cue trading), and word choice (Pickering and Greene, 2019; Jackson Hearns and Kremer, 2018; Mills and Stoneham, 2017). Typically, the voice is perceived as more masculine when associated with lower pitches and darker timbres, and more feminine when associated with higher pitches and brighter timbres. But this is not to say that both cues are independent (Krumhansl, 1990; Beal, 1985). There is some evidence that pitch and timbre (and loudness to some degree) can often be conflated when humans attempt to classify sounds (Allen and Oxenham, 2014; Krumhansl and Iverson, 1992; Melara and Marks, 1990). High-frequency sounds are often perceived as bright in timbre and low-frequency sounds dark in timbre. This appears to be what the ear expects and implies that a form of incongruency occurs when high-pitched sounds are combined with dark timbres, and vice-versa. Whether these interactions (i.e. timbre's influence on pitch perception tasks, or pitch's influence of timbre perception tasks) are symmetric or asymmetric is not well agreed upon. Discrepancies among studies which specifically examined this point are likely due to methodological differences, e.g., whether the salience of the distracting cue was controlled or not (Allen and Oxenham, 2014). Also, perhaps surprisingly, musicians are only slightly better than non-musicians at mitigating this confusion, suggesting that it is very difficult to perceptually isolate one cue over the other (Allen and Oxenham, 2014).

The salience of pitch and timbre aspects in the voice is already challenging to control for, experimentally (e.g., with physical changes of the fundamental frequency or vocal tract length), but determining the objective meaning of the terminology and semantics that people use when internally appraising a voice is even harder. Between pitch and timbre, pitch is perhaps the more salient of the two dimensions (Krumhansl, 1990), but we would offer that we are better trained to describe its dimension since it is a simpler construct referred to as “low” vs. “high” (American National Standards Institute, 1994). Timbre, on the other hand, is a complex construct, which lacks a targeted vocabulary and is subject to prior experience (Saitis and Weinzierl, 2019). It is fair to state that overall, timbre remains a misunderstood attribute of sound (Aaen et al., 2024; Siedenburg and McAdams, 2017). Following on the third (collective) world view, it is likely that vocal timbre is created by the listener as much as by the speaker (due to its cultural connections and shared timbre semantics). People tend to assume that bright voices will also be high and small voices, while dark voices will be low and big, but this is not always the case. It is easy to observe these errors in judgment through the inhalation of helium and sulfur hexafluoride. With inhaled helium, pitch is perceived as having gone up, even when it remains constant, because the upper harmonics are emphasized (Wentworth, 2011). With the inhalation of sulfur hexafluoride, pitch is perceived as having gone down because the lower harmonics are emphasized (Stiefel, 2018). That is, the length and mass of the vocal folds do not change with inhalation of these gasses, meaning at the sound source, the speaker can still only produce the frequencies they could produce prior to inhaling the gasses. This perhaps points to the fact that vocal timbre is more complicated than instrumental timbre, because voices can be almost infinitely varied by the trained user (Stone and Erickson, 2023; Noble, 2022; Erickson, 2016; Town and Bizley, 2013) whereas instrumental timbre is more consistent across instruments and players (Heidemann, 2016). Given that timbre is a strong marker for gender, perhaps stronger than pitch (Pernet and Belin, 2012; Rendall et al., 2005; Titze, 1994), it is a cue that trans individuals might have focussed on and with which they have developed expertise.

### 1.3 Hypotheses

The goal of the present study was to once again explore the perception of voices by cis and trans participants. In contrast to our previous study using the original recordings of 18 actors, here we manipulated (through digital signal processing) the F0 (experiment 1) or the VTL (experiment 2) of the same actors, leaving the video content intact (no visual incongruency as in the McGurk effect). If the trans advantage was primarily due to ignoring visual information, we expected to simply replicate this advantage regardless of the manipulation. If, instead, the trans advantage was somewhat related to better processing of auditory aspects of the manipulated voices, we should observe differences between the two manipulations. The specific hypotheses were:

(1) In experiment 1, even though listeners could rely on the VTL of each actor (left intact), we hypothesized that manipulating the F0 by +/−3 semitones (a difference easily detectable, e.g., see Moore and Glasberg, 1990) would affect the appraisal of A-alone stimuli with respectively higher and lower ratings. (2) In experiment 2, even though listeners could rely on the F0 of each actor (left intact), we hypothesized that manipulating the VTL by +/−3 semitones^1^ (a difference easily detectable, Gaudrain and Başkent, 2015) would affect the appraisal of A-alone stimuli with respectively darker and brighter ratings. These two predictions essentially rely on the assumption that listeners' judgment of voices is always influenced by pitch and timbre together and cannot be made on a single cue exclusively, in line with the idea that both cues are difficult to perceptually disentangle (Allen and Oxenham, 2014). Finally (3) more to the point of this study, we expected that the AV ratings would differ from A ratings, and specifically in the direction of V ratings and (4) we hypothesized this AV shift to be smaller in trans participants than in cis participants, and perhaps particularly reduced for the cue to which trans participants happen to be more sensitive. And given the cultural traits of timbre, we suspected that it could represent a more malleable cue for trans participants to use.

## 2 Methods

### 2.1 Participants

**Figure 1:**
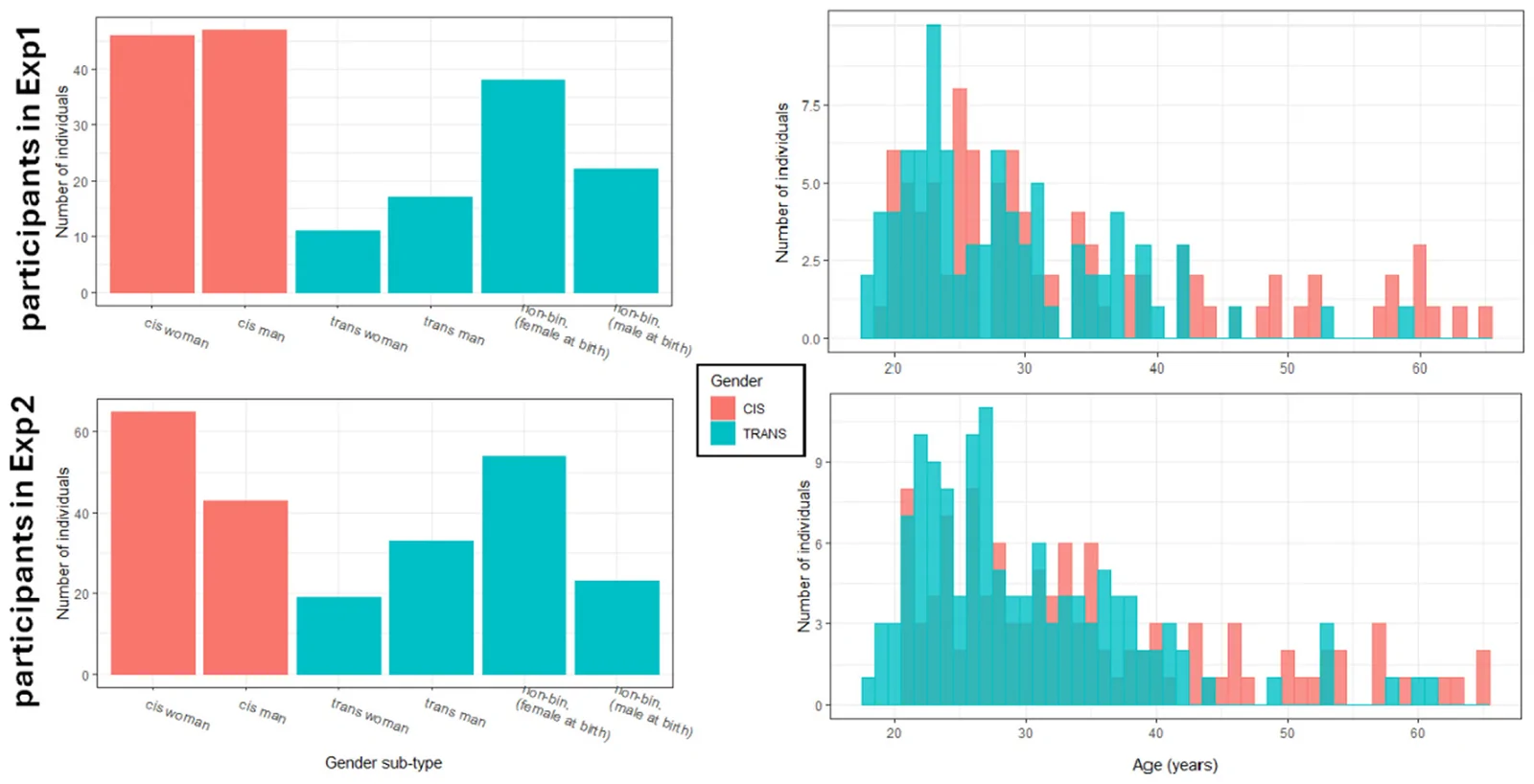
Demographics of the two groups of participants, split by sub-type of gender identity (**left** panels), and age at time of testing (**right** panels), in experiment 1 **(top)** and experiment 2 **(bottom)**.

In our previous study, the trans advantage was observed with samples of 85 cis and 81 trans individuals. Simply assuming a similar effect size here, we aimed for a similar (or larger) sample size. In Experiment 1, 416 participants were recruited initially on the Prolific platform (https://www.prolific.com/) in two batches. The first batch consisted of gender expansive individuals, between the ages of 18 and 70. Within this sample, there were four sub-groups: transgender men, transgender women, non-binary people assigned male at birth (NB-AMAB), and non-binary people assigned female at birth (NB-AFAB), here referred to (for clarity) under the umbrella term TRANS. The second batch consisted of cisgender men and cisgender women, between the ages of 18 and 70, referred to as CIS. Many participants either had technical difficulties or quit study before completing the study because the AV materials took about 15 min to download. The incomplete files were automatically excluded from any analysis. In the remaining files, 88 were in the TRANS group and 93 were in the CIS group (Figure 1, top-left). Similarly, in experiment 2, 436 participants were initially recruited on Prolific platform, but after rejection of the participants who quit, there were 129 in the TRANS group, and 108 in the CIS group (Figure 1, bottom-left).

The two groups were matched in most variables, except age. This happened in experiment 1 [*t*(179) = 2.7, *p* = 0.008] and in experiment 2 [*t*(235) = 3.5, *p* < 0.001] where the CIS group was a bit older than the TRANS group (33.0 vs. 28.7 years, and 34.5 vs. 30.0 years, right panels in Figure 1). This difference was unfortunate but presumably reflects a genuine difficulty in recruiting trans people online beyond 50 years of age. Note that this did not occur in our previous study where all participants were younger than 32 years of age. While it may have been a more judicious choice for group comparison, here we thought it was interesting to broaden our inquiry toward older samples who tend to have more binary views (including on gender roles in society). To explore this potential confound, we made a few attempts to narrow the groups using the MatchIt package (Ho et al., 2011) so that age was not significantly different between the two groups; this would for example have resulted in removing 12 CIS participants and 3 TRANS participants in experiment 1, or 12 CIS participants and 6 TRANS participants in experiment 2, but the pattern of findings was unchanged. So, we decided not to proceed with this route and simply acknowledge that age was a potential caveat in this study (see further discussion in the Limitation section).

### 2.2 Stimuli

Stimuli came from the Ryerson Audio-Visual Database of Speech and Song (RAVDESS), freely available online (Livingstone and Russo, 2018). While these materials were created for emotion perception research, we did not exploit it for this purpose but simply because it offered a diversity of actors and has been analyzed acoustically (e.g., all speech stimuli were examined in Major and Chatterjee, 2026; and the sung stimuli were described in the original paper). A subset of 18 actors (from the original 24) was selected as in our previous study, with sung and spoken utterances (either “kids are talking by the door” or “dogs are sitting by the door”), all enacted with neutral emotion. There were as many sung as spoken stimuli, and the two sentences were equally represented in each mode. There were three actors from each voice category (bass, baritone, tenor, alto, mezzo, and soprano). All stimuli existed in an audio-alone (A) version (resolution of 16 bit, and sampled at 48 kHz), a visual-alone (V) version containing no sound and never manipulated, and a combined audiovisual (AV) version. We used the software Straight to alter audio stimuli by plus and minus 3 semitones in F0 while not altering the VTL (experiment 1), and used Praat to alter the audio stimuli by plus and minus 3 semitones in VTL while not altering the F0 (experiment 2). The audio content was then reintegrated seamlessly into the videos using Adobe and appeared natural as no temporal alteration was made to the audio. To clarify, these manipulations were expected to shift A ratings systematically (i.e., a higher F0 or shorter VTL being perceived as higher/brighter voice, regardless of the actor) but not shift AV ratings systematically (because a higher F0 or shorter VTL would push basses/baritones/tenors toward more androgynous voices, while pushing altos/mezzos/sopranos toward more gender-conforming voices; and vice-versa). We therefore ended up with 144 stimuli in each experiment: 72 stimuli with F0 shifted upward and 72 shifted downward, in experiment 1; and 72 stimuli with VTL shifted downward and 72 shifted upward, in experiment 2. To clarify, the 72 stimuli resulted from 18 actors × 2 modes (spoken or sung) × 2 sentences. All the resulting videos appeared very natural, largely devoid of acoustic artifacts, but note that participants were never presented with the unaltered materials from the previous paper (Marchand Knight et al., 2023).

### 2.3 Design and procedure

As a first step, participants answered questions on demographics related to age and gender identity. Although this information was collected on Prolific, we wanted participants to provide their own gender identification at the time of testing as these identities may be nuanced or fluid throughout life (and thus have changed since the date of their Prolific account creation). Next, participants completed a questionnaire of gender roles in society (see the full questionnaire in Supplementry Material 5 in Marchand Knight et al., 2023 or see Tezare, 2015).

Then, participants completed a short tutorial in which they heard all 6 voice types (bass, baritone, tenor, alto, mezzo, and soprano) in A-only versions, in both spoken and sung modalities. They were told the correct voice category and trained (with feedback) to move a slider along a continuous spectrum labeled low/dark to high/bright. The use of a continuous response scale was meant to discourage participants from thinking of the voice in binary terms.

In the three test blocks, participants were always asked to appraise stimuli (whether they were A, V, or AV) on a continuous slider from low/dark to high/bright, with the 6 voice types written underneath the scale (bass, baritone, tenor, alto, mezzo, soprano) equally spaced to avoid creating a gender break. In other words, the paradigm was similar to a voice classification task except we never attempted to discretize each answer into a box. Each rating (the dependent variable) was a continuous number from 1 to 6. In block 1, participants rated the 144 A-only stimuli. In block 2, they rated the 144 V-only stimuli. In block 3, they rated the 144 AV combinations. The blocks were always presented in the A, V, AV order (such that participants would never associate a physical appearance with a voice until the third block), while the order of stimuli within each block was randomly shuffled, differently for each block and each participant. The analysis focused on the relationship of each participant's rating between the three modalities. That is, we wanted to see if a participant's rating of the voice would change once they were confronted with the actor's physical appearance.

Finally, just before exiting the study, participants answered questions regarding technical difficulties, mental effort, intelligibility of the instructions and training, perceived goal of the study, and the quality of the stimuli. The answers were reviewed but did not provide much insight and are not reported in the rest of this article. Participants gave informed consent in keeping with the rules of Concordia University's Institutional Review Board, and they received $10 for their participation.

### 2.4 Equipment

The two experiments were run fully online, with no direct control over participants' audio equipment. There was no difference between the CIS and TRANS groups in the type of audio device used [χ^2^(3, *N* = 181) = 0.2, *p* = 0.969, in experiment 1; χ^2^(3, *N* = 237) = 6.5, *p* = 0.088, in experiment 2]: about 37% of participants used headphones, 20% used external speakers, 21% used earbuds, and 22% used their default laptop output. Participants were instructed to set their sound at a comfortable level during practice and not change it afterwards.

## 3 Data Analysis

### 3.1 Ratings for A-only and V-only stimuli

To assess whether our manipulations had the desired effect, we examined the pattern of ratings in each modality alone. A repeated-measures analysis of variance (rm-ANOVA) was conducted on the A-only and V-only ratings with one between-subject factor: participant gender (CIS vs. TRANS), and three within-subject factors: (1) actor category (6 levels: bass, baritone, tenor, alto, mezzo, and soprano), (2) mode of stimuli (2 levels: spoken or sung), and (3) the manipulation (2 levels: high vs. low F0 in experiment 1, or short vs. long VTL in experiment 2). Degrees of freedom were corrected with Greenhouse-Geisser adjustments, necessary for all effects that involved actor category as the assumption of sphericity was always violated.

### 3.2 AV shifts

For each of the 144 AV stimuli, we extracted the (AV–A) difference and multiplied it by the sign of the (V–A) difference, such that a positive value always indicated a shift of the AV appraisal toward the V rating. For example, an AV rating could be 4.7 (on the 6-point scale) while the A rating could be 5.2; this would be an AV shift of 0.5 in the direction of the V rating (for example 4.5), so we assign it a positive sign: here +0.5. This was the key metric to determine whether an individual was or was not subject to visual bias. Again, a rm-ANOVA was conducted with the same four factors (gender, actor category, mode, and the F0/VTL manipulation).

### 3.3 Gender views

For each question in the questionnaire on gender views, there were 4 possible choices: 1 for “completely disagree,” 2 for “somewhat disagree,” 3 for “somewhat agree,” and 4 for “completely agree.” These ordinal data were examined with a generalized linear model using a binomial distribution. Additional analyses were conducted to see whether these gender views related to the AV shift. In each experiment, a linear regression was performed between each participant's average score from the questionnaire and their averaged AV shift.

## 4 Results

### 4.1 Experiment 1: F0 manipulation

**Figure 2:**
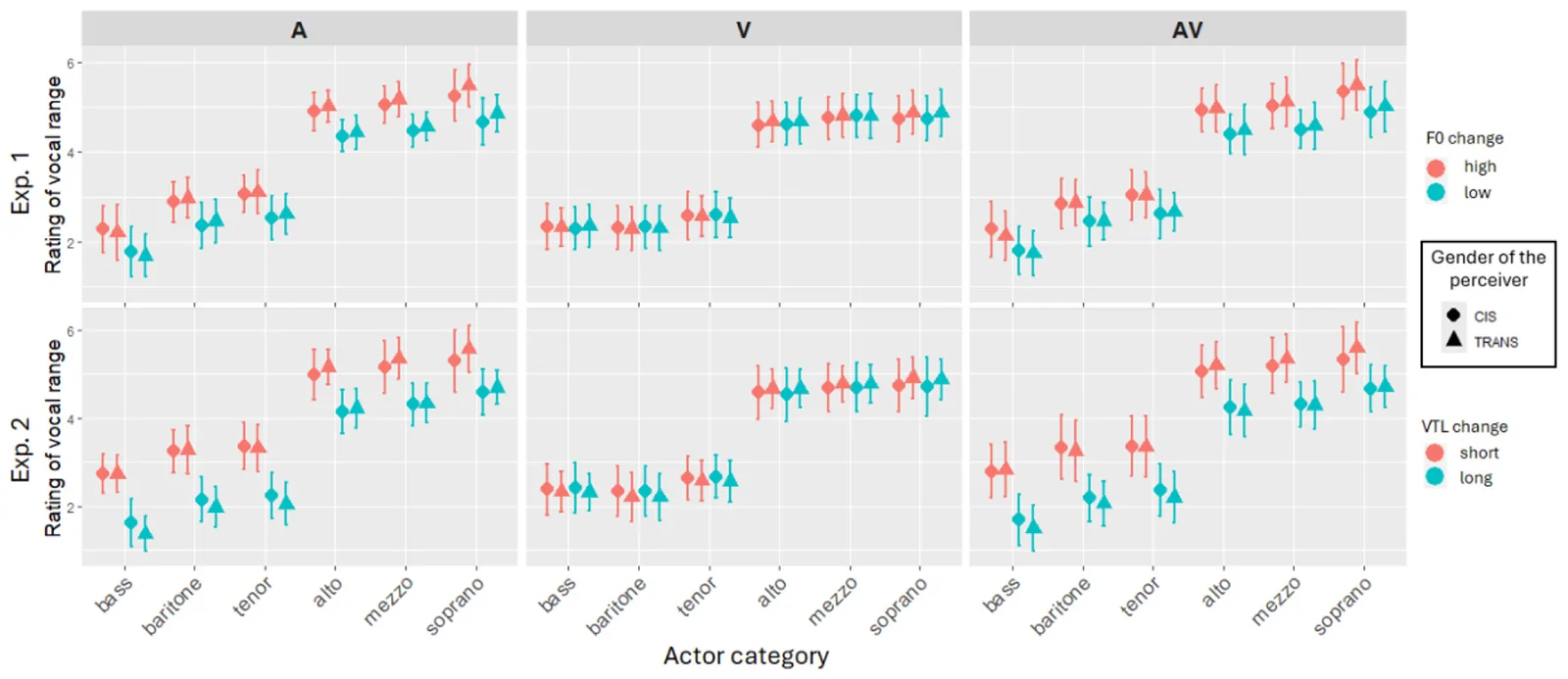
Mean ratings of six categories of actors, manipulated with lower F0 in blue or higher F0 in red **(top)** or manipulated with longer VTL in blue or shorter VTL in red **(bottom)**, conducted by cis (circles) and trans (triangles) participants, listening to A stimuli alone **(left)**, watching the V silently **(middle)**, or with both A and V **(right)**. Error bars show one standard deviation.

**Figure 3:**
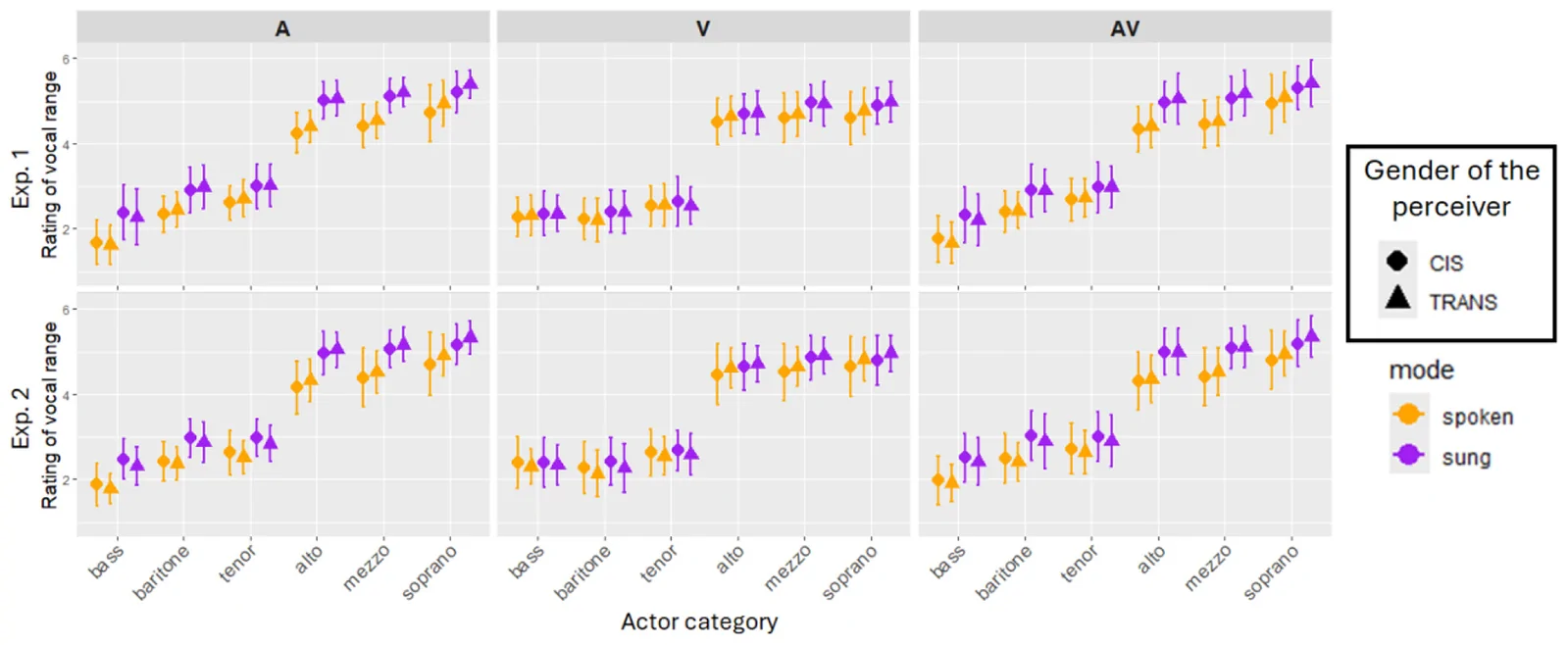
Same as Figure 2 but with mode (spoken/sung) instead of the F0/VTL manipulation.

First, we report on the analyses of the A-alone ratings which did vary systematically with the F0 manipulation, and the V-alone ratings which did not (since the visual content was indeed unchanged). Both are shown in Figures 2, 3 (top left and middle panels). The pattern of findings was overall complex (but expected), so we present it in order of relevance to the current topic, i.e., with anything related to the manipulation of interest first, followed by other observations already reported in Marchand Knight et al. (2023). The full statistical results are shown in Table 1.

**Table 1:** Statistical results of the 4-way ANOVA conducted in each experiment for three dependent variables, the A-only rating, the V-only rating, and the AV shift.

Independent variable	Experiment 1 (F0 manipulations)			Experiment 2 (VTL manipulations)		
Gender	F(1,179) = 6.4, *p* = 0.012, $\eta_{\text{p}}^{2}$ = 0.035	F(1,179) = 1.1, *p* = 0.290, $\eta_{\text{p}}^{2}$ = 0.006	F(1,179) < 0.1, *p* = 0.954, $\eta_{\text{p}}^{2}<$0.001	F(1,235) = 0.2, *p* = 0.631, $\eta_{\text{p}}^{2}$ < 0.001	F(1,235) < 0.1, *p* = 0.766, $\eta_{\text{p}}^{2}$ < 0.001	F(1,235) = 8.4, *p* = 0.004, $\eta_{\text{p}}^{2}$ = 0.035
Manipulation (F0/VTL)	F(1,179) = 888.5, *p* < 0.001, $\eta_{\text{p}}^{2}$ = 0.832	F(1,179) = 0.5, *p* = 0.500, $\eta_{\text{p}}^{2}$ = 0.003	F(1,179) = 3.6, *p* = 0.058, $\eta_{\text{p}}^{2}$ = 0.020	F(1,235) = 1252.1, *p* < 0.001, $\eta_{\text{p}}^{2}$ = 0.842	F(1,235) = 0.4, *p* = 0.549, $\eta_{\text{p}}^{2}$ = 0.002	F(1,235) = 14.9, *p* < 0.001, $\eta_{\text{p}}^{2}$ = 0.060
Category (of actors)	F(1.5, 262.9) = 2021, *p* < 0.001, $\eta_{\text{p}}^{2}$ = 0.919	F(1.8, 328.8) = 1439, *p* < 0.001, $\eta_{\text{p}}^{2}$ = 0.889	F(3.6, 637.4) = 1.3, *p* = 0.265, $\eta_{\text{p}}^{2}$ = 0.007	F(1.6, 366.3) = 3449, *p* < 0.001, $\eta_{\text{p}}^{2}$ = 0.936	F(1.9, 455.1) = 1838, *p* = 0.001, $\eta_{\text{p}}^{2}$ = 0.887	F(4.4, 1039.6) = 4.3, *p* = 0.001, $\eta_{\text{p}}^{2}$ = 0.018
Mode	F(1,179) = 438.9, *p* < 0.001, $\eta_{\text{p}}^{2}$ = 0.710	F(1,179) = 76.9, *p* < 0.001, $\eta_{\text{p}}^{2}$ = 0.300	F(1,179) = 0.4, *p* = 0.540, $\eta_{\text{p}}^{2}$ = 0.002	F(1,235) = 449.0, *p* < 0.001, $\eta_{\text{p}}^{2}$ = 0.656	F(1,235) = 55.3, *p* < 0.001, $\eta_{\text{p}}^{2}$ = 0.190	F(1,235) = 4.7, *p* = 0.031, $\eta_{\text{p}}^{2}$ = 0.020
Gender × Manipulation	F(1,179) < 0.1, *p* = 0.868, $\eta_{\text{p}}^{2}$ < 0.001	F(1,179) = 0.9, *p* = 0.349, $\eta_{\text{p}}^{2}$ = 0.005	F(1,179) = 0.1, *p* = 0.747, $\eta_{\text{p}}^{2}$ < 0.001	F(1,235) = 9.2, *p* = 0.003, $\eta_{\text{p}}^{2}$ = 0.037	F(1,235) = 0.3, *p* = 0.608, $\eta_{\text{p}}^{2}$ = 0.001	F(1,235) = 0.8, *p* = 0.387, $\eta_{\text{p}}^{2}$ = 0.003
Gender × Category	F(1.5, 262.9) = 2.5, *p* = 0.103, $\eta_{\text{p}}^{2}$ = 0.014	F(1.8, 328.8) = 0.9, *p* = 0.404, $\eta_{\text{p}}^{2}$ = 0.005	F(3.6, 637.4) = 1.1, *p* = 0.363, $\eta_{\text{p}}^{2}$ = 0.006	F(1.6, 366.3) = 10.1, *p* < 0.001, $\eta_{\text{p}}^{2}$ = 0.041	F(1.9, 455.1) = 4.6, *p* = 0.011, $\eta_{\text{p}}^{2}$ = 0.019	F(4.4, 1039.6) = 3.7, *p* = 0.004, $\eta_{\text{p}}^{2}$ = 0.015
Gender × Mode	F(1,179) = 1.3, *p* = 0.259, $\eta_{\text{p}}^{2}$ = 0.007	F(1,179) = 4.5, *p* = 0.036, $\eta_{\text{p}}^{2}$ = 0.024	F(1,179) = 0.4, *p* = 0.536, $\eta_{\text{p}}^{2}$ = 0.002	F(1,235) = 1.0, *p* = 0.307, $\eta_{\text{p}}^{2}$ = 0.004	F(1,235) = 0.5, *p* = 0.458, $\eta_{\text{p}}^{2}$ = 0.002	F(1,235) = 0.2, *p* = 0.631, $\eta_{\text{p}}^{2}$ < 0.001
Manipulation × Category	F(4.3, 777.8) = 3.6, *p* = 0.005, $\eta_{\text{p}}^{2}$ = 0.020	F(4.7, 839.0) = 0.4, *p* = 0.805, $\eta_{\text{p}}^{2}$ = 0.002	F(4.6, 830.1) = 1.5, *p* = 0.185, $\eta_{\text{p}}^{2}$ = 0.008	F(3.2, 751.7) = 55.9, *p* < 0.001, $\eta_{\text{p}}^{2}$ = 0.192	F(4.8, 1118.0) = 1.0, *p* = 0.414, $\eta_{\text{p}}^{2}$ = 0.004	F(4.2, 985.2) = 4.7, *p* < 0.001, $\eta_{\text{p}}^{2}$ = 0.020
Manipulation × Mode	F(1,179) < 0.1, *p* = 0.921, $\eta_{\text{p}}^{2}$ < 0.001	F(1,179) = 0.5, *p* = 0.469, $\eta_{p}^{2}=$0.003	F(1,179) = 6.6, *p* = 0.011, $\eta_{\text{p}}^{2}$ = 0.036	F(1,235) = 49.2, *p* < 0.001, $\eta_{\text{p}}^{2}$ = 0.173	F(1,235) < 0.1, *p* = 0.907, $\eta_{\text{p}}^{2}$ < 0.001	F(1,235) = 15.2, *p* < 0.001, $\eta_{\text{p}}^{2}$ = 0.061
Category × Mode	F(3.5, 621.0) = 34.2, *p* < 0.001, $\eta_{\text{p}}^{2}$ = 0.161	F(3.8, 675.8) = 25.1, *p* < 0.001, $\eta_{\text{p}}^{2}$ = 0.123	F(4.6, 823.9) = 1.5, *p* = 0.205, $\eta_{\text{p}}^{2}$ = 0.008	F(3.3, 772.2) = 39.5, *p* < 0.001, $\eta_{\text{p}}^{2}$ = 0.144	F(4.4, 1022.6) = 29.7, *p* < 0.001, $\eta_{\text{p}}^{2}$ = 0.112	F(4.7, 1107.8) = 1.9, *p* = 0.089, $\eta_{\text{p}}^{2}$ = 0.008
Gender × Mani*p*ulation × Category	F(4.3, 777.8) = 0.6, *p* = 0.692, $\eta_{\text{p}}^{2}$ = 0.003	F(4.7, 839.0) = 1.9, *p* = 0.094, $\eta_{\text{p}}^{2}$ = 0.011	F(4.6, 830.1) = 1.2, *p* = 0.293, $\eta_{\text{p}}^{2}$ = 0.007	F(3.2, 751.7) = 1.0, *p* = 0.417, $\eta_{\text{p}}^{2}$ = 0.004	F(4.8, 1118.0) = 1.2, *p* = 0.331, $\eta_{\text{p}}^{2}$ = 0.005	F(4.2, 985.2) = 1.4, *p* = 0.217, $\eta_{\text{p}}^{2}$ = 0.006
Gender × Manipulation × Mode	F(1,179) < 1.9, *p* = 0.170, $\eta_{\text{p}}^{2}$ = 0.010	F(1,179) = 0.8, *p* = 0.370, $\eta_{\text{p}}^{2}$ = 0.004	F(1,179) = 1.9, *p* = 0.172, $\eta_{\text{p}}^{2}$ = 0.010	F(1,235) = 0.4, *p* = 0.516, $\eta_{\text{p}}^{2}$ = 0.002	F(1,235) = 0.8, *p* = 0.377, $\eta_{\text{p}}^{2}$ = 0.003	F(1,235) < 0.1, *p* = 0.825, $\eta_{\text{p}}^{2}$ < 0.001
Gender × Category × Mode	F(3.5, 621.0) = 0.2, *p* = 0.907, $\eta_{\text{p}}^{2}$ = 0.001	F(3.8, 675.8) = 1.4, *p* = 0.227, $\eta_{\text{p}}^{2}$ = 0.008	F(4.6, 823.9) = 1.6, *p* = 0.159, $\eta_{\text{p}}^{2}$ = 0.009	F(3.3, 772.2) = 0.2, *p* = 0.881, $\eta_{\text{p}}^{2}$ = 0.001	F(4.4, 1022.6) = 1.9, *p* = 0.096, $\eta_{\text{p}}^{2}$ = 0.008	F(4.7, 1107.8) = 0.8, *p* = 0.511, $\eta_{\text{p}}^{2}$ = 0.004
Mani*p*ulation × Category × Mode	F(4.7, 841.0) = 17.9, *p* < 0.001, $\eta_{\text{p}}^{2}$ = 0.091	F(4.8, 852.0) = 0.3, *p* = 0.888, $\eta_{\text{p}}^{2}$ = 0.002	F(5,895) = 0.6, *p* = 0.664, $\eta_{\text{p}}^{2}$ = 0.004	F(4.5, 1049.5) = 15.7, *p* < 0.001, $\eta_{\text{p}}^{2}$ = 0.063	F(4.6, 1084.8) = 1.8, *p* = 0.113, $\eta_{\text{p}}^{2}$ = 0.008	F(4.8, 1127.5) = 3.2, *p* = 0.008, $\eta_{\text{p}}^{2}$ = 0.013
4-way	F(4.7, 841.0) = 0.7, *p* = 0.611, $\eta_{\text{p}}^{2}$ = 0.004	F(4.8, 852.0) = 0.5, *p* = 0.734, $\eta_{\text{p}}^{2}$ = 0.003	F(5,895) = 0.8, *p* = 0.580, $\eta_{\text{p}}^{2}$ = 0.004	F(4.5, 1049.5) = 1.2, *p* = 0.318, $\eta_{\text{p}}^{2}$ = 0.005	F(4.6, 1084.8) = 1.0, *p* = 0.413, $\eta_{\text{p}}^{2}$ = 0.004	F(4.8, 1127.5) = 1.7, *p* = 0.125, $\eta_{\text{p}}^{2}$ = 0.007

The main manipulation was F0 (in experiment 1) or VTL (in experiment 2) with 2 levels (high/bright vs. low/dark). The mode (2 levels: spoken or sung) and the actor category (6 levels: bass, baritone, tenor, alto, mezzo, soprano) were two other within-subject factors while group (gender: CIS vs. TRANS) was a between-subject factor.

#### 4.1.1 A-ratings

There was a main effect of F0 manipulation [*p*<*0.001*]: marginal estimates were 3.949 [3.913–3.985] for the high F0, and 3.397 [3.361–3.433] for the low F0, on average across actors. Given that participants only used about 3 units (instead of the 6 available) between the extremities of our actor categories (from about 2 in basses to 5 in sopranos), this effect of +0.552 is not negligible, and is comparable to one change in actor category. This F0 effect was somewhat different across the six actor categories [*p* = *0.005*], respectively +0.513, +0.514, +0.520, +0.564, +0.602, +0.597 for bass, baritone, tenor, alto, mezzo, and soprano. It also depended on mode but not to the same extent across categories resulting in a 3-way interaction [*p*<*0.001*]: estimates were +0.537, +0.613, +0.611, +0.453, +0.530, and +0.570 when each of the six actors spoke compared to +0.488, +0.413, +0.429, +0.674, +0.674, and +0.624 when each of the six actors sang. It is interesting to note that the F0 manipulation had more impact when male voices spoke than when they sang, whereas it had more impact when female voices sang than when they spoke. There was a main effect of gender [*p* = *0.012*], which was not particularly relevant on its own (simply reflecting a slightly different location of average rating on the response scale), but gender did not interact with F0 [*F(1,179)*<*0.1, p* = *0.868*] suggesting that *this manipulation affected both groups equally*. Also, gender did not interact with any other factor or combination of factors [*p* > *0.103*].

Other effects or interactions were expected from Marchand Knight et al. (2023). Namely, there was a strong effect of voice category [*p*<*0.001*] reflecting that listeners allocated actors to each category in the correct order. Marginal estimates were 1.985 [1.924– 2.047] for bass, 2.669 [2.608–2.730] for baritone, 2.828 [2.767–2.889] for tenor, 4.677 [4.616–4.739] for alto, 4.815 [4.754–4.876] for mezzo, and 5.065 [5.003–5.126] for soprano. A gap is notable between the bottom three (traditionally male) and the top three (traditionally female) categories, suggesting that participants are still instilled with binary views of voices and do not report voices simply on a diagonal. There was a main effect of mode [*p*<*0.001*] reflecting higher ratings in sung than in spoken stimuli. Marginal estimates were 3.961 [3.920–4.002] for sung stimuli and 3.386 [3.345–3.427] for spoken stimuli. Furthermore, this mode difference was itself more pronounced for some categories than others, resulting in an interaction between mode and category [*F(3.5,621.0)* = *34.2, p*<*0.001*].

#### 4.1.2 V-ratings

The F0 manipulation did not have any effect [*p* = *0.500*] nor did it interact with anything else [*p* > *0.094*], which is reassuring since the video content was precisely identical. Any other effect or interaction was expected from our previous study. The main effect of voice category [*p*<*0.001*] reflected that participants made a guess on the actors' voice type (exclusively based on the actors' face and body size/shape). This guess was binary: marginal estimates were 2.325 [2.257–2.394] for bass, 2.306 [2.238–2.375] for baritone, 2.565 [2.497–2.634] for tenor, and elevated to 4.645 [4.577–4.714] for alto, 4.790 [4.722–4.859] for mezzo, and 4.805 [4.737–4.874] for soprano. The main effect of mode was significant [*p*<*0.001*] reflecting higher ratings in sung than in spoken stimuli. Marginal estimates were 3.653 [3.617–3.690] for sung stimuli, and 3.492 [3.456–3.529] for spoken stimuli. This mode difference was more pronounced for mezzos than other categories (respectively +0.059, +0.180, +0.038, +0.137, +0.303, +0.249 for bass, baritone, tenor, alto, mezzo, and soprano), resulting in an interaction between mode and category [*p*<*0.001*]. Gender did not result in a main effect [*p* = *0.290*], but interacted with mode [*p* = *0.036*]: the mode effect was larger in the CIS group (+0.200) than in the TRANS group (+0.122). No other main effect or interaction was significant.

#### 4.1.3 AV-ratings

**Figure 4:**
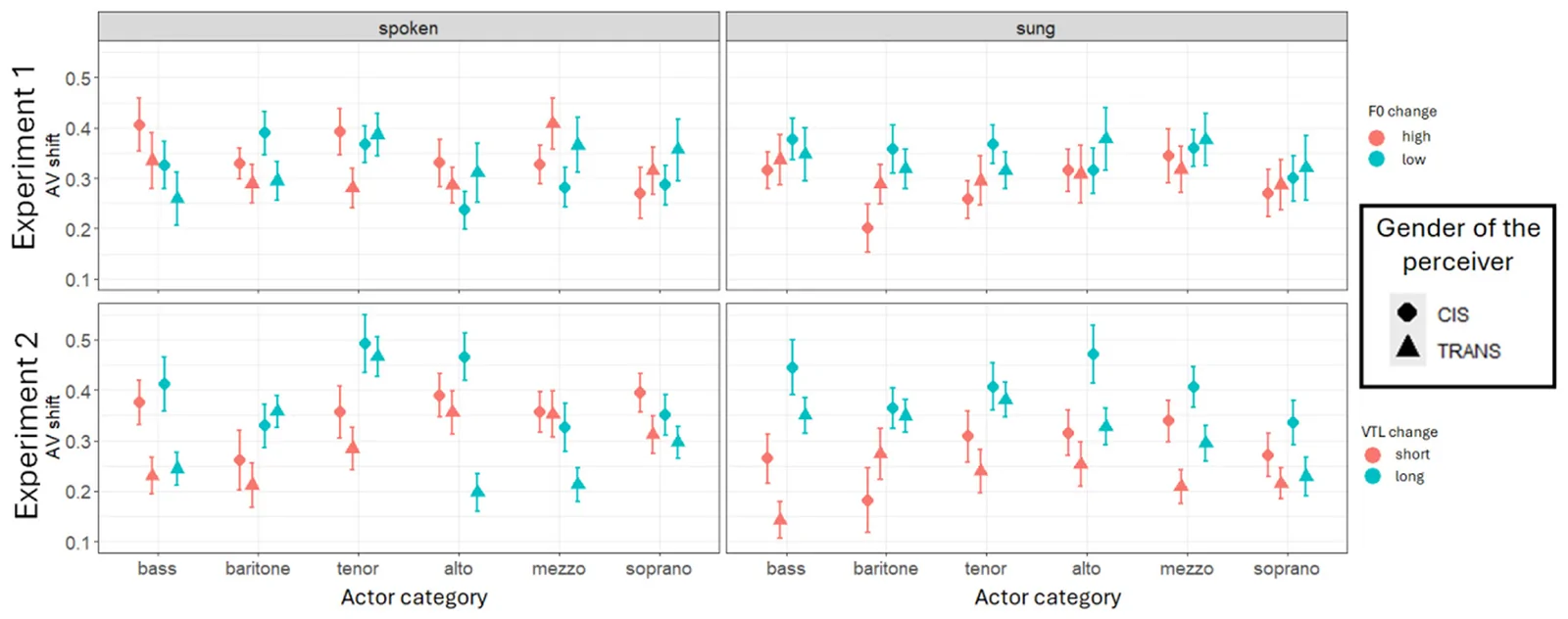
Audiovisual shifts obtained in experiment 1 **(top)** and experiment 2 **(bottom)**, with spoken **(left)** and sung **(right)** stimuli, calculated from the difference (AV–A) ratings multiplied by the sign of (V–A) shift so that a positive value always indicates a shift toward the visual influence. Error bars show one standard error of the mean.

As expected, AV ratings were systematically shifted away from A ratings, in the direction of V ratings. This is not obvious in Figure 2 or Figure 3 because the upward shifts are counteracted by the downward shifts (and the experiment was designed to have a roughly equal representation of both cases). But once they were multiplied (by the sign of V–A) in Figure 4 (top panels), they were significantly above 0 [*t(180)* > *9.6, p*<*0.001 in all cases*]. In other words, participants were *systematically biased in their appraisal of voices by the actors' physical appearance and gestures*. As a novel finding: the main effect of F0 manipulation did not reach significance [*p* = *0.058*] but it interacted with mode [*p* = *0.011*]. Indeed, the manipulation only had an effect with sung stimuli [*p* = *0.002*], not with spoken stimuli [*p* = *0.563*] (top-right vs. top-left, Figure 4). This result suggested that voices sung with lower F0 suffered from more visual bias against them than voices sung with higher F0 (0.344 vs. 0.295). Any other effect or interaction was not significant [*p* > *0.159*]. Importantly, TRANS individuals did not resist the bias compared to CIS individuals [*p* = *0.954*] in striking contrast to our earlier observation (Marchand Knight et al., 2023).

### 4.2 Experiment 2: VTL manipulation

First, we report on the analyses of the A-alone ratings which did vary systematically with the VTL manipulation, and the V-alone ratings which did not. Both are shown in Figures 2, 3 (bottom left and middle panels). The pattern of findings is again presented in order of relevance to the current topic, i.e., with anything related to the manipulation of interest first, followed by other observations already reported in Marchand Knight et al. (2023). The full statistical results are shown in Table 1.

#### 4.2.1 A-ratings

There was a main effect of VTL manipulation [*p*<*0.001*]: marginal estimates were 4.180 [4.136–4.225] for short VTLs, and 3.142 [3.098–3.186] for long VTL, on average across actors. Note that this effect (+1.038) is substantial (equivalent to changes of about 2 categories). This VTL induced difference was somewhat different across the six actor categories [*p*<*0.001*], respectively +1.238, +1.199, +1.194, +0.880, +0.931, +0.789 for bass, baritone, tenor, alto, mezzo, soprano. It also depended on mode [*p*<*0.001*]: its effect was slightly larger when actors spoke (+1.110) than when actors sang (+0.967), and this interaction was more or less observable across the six categories resulting in a 3-way interaction [*p*<*0.001*]. Estimates were +1.236, +1.408, +1.301, +0.908, +0.953, and +0.856 when each of the six actors spoke compared to +1.239, +0.991, +1.088, +0.853, +0.908, and +0.723 when each of the six actors sang. Apart from basses, the VTL manipulation had more impact when actors spoke than when they sang. Critically, gender did not result in a main effect [*p* = *0.631*] but it interacted with VTL [*p* = *0.003*] because the manipulation had stronger effect in TRANS (+1.127) than in CIS (+0.950) listeners. This important finding suggests that trans individuals *are more sensitive than cis listeners to the timbre manipulation*.

Other effects or interactions were expected from our previous study. Namely, there was a main effect of voice category [*p*<*0.001*] reflecting that listeners allocated actors to each category in the correct order. Marginal estimates were 2.116 [2.064–2.168] for bass, 2.662 [2.610–2.713] for baritone, 2.743 [2.691–2.795] for tenor, 4.627 [4.575–4.679] for alto, 4.786 [4.735–4.838] for mezzo, and 5.033 [4.981–5.085] for soprano. The gap between the bottom three and the top three categories was replicated. There was a main effect of mode [*p*<*0.001*] reflecting higher ratings in sung than in spoken stimuli. Marginal estimates were 3.936 [3.894–3.978] for sung stimuli and 3.387 [3.345– 3.429] for spoken stimuli. Furthermore, this mode difference was more pronounced for some categories than others, resulting in an interaction between mode and category [*p*<*0.001*]. Gender also interacted with voice category [*p*<*0.001*] because the TRANS group tended to use a broader range of ratings than the CIS group.

#### 4.2.2 V-ratings

The VTL manipulation did not have any effect [*p* = *0.549*] nor did it interact with anything else [*p* > *0.106*], which once again is reassuring since the video content was precisely identical. Any other effect or interaction were expected. The main effect of voice category [*p*<*0.001*] reflected that participants made a guess on the actors' voice type (exclusively based on the actors' face and body size/shape). This guess was binary: marginal estimates were 2.358 [2.294–2.422] for bass, 2.269 [2.205–2.333] for baritone, 2.610 [2.547–2.674] for tenor, and elevated to 4.608 [4.545–4.672] for alto, 4.733 [4.670–4.797] for mezzo, and 4.809 [4.745–4.873] for soprano. The main effect of mode was significant [*p*<*0.001*] reflecting higher ratings in sung than in spoken stimuli. Marginal estimates were 3.629 [3.589–3.669] for sung stimuli, and 3.500 [3.461–3.540] for spoken stimuli. This mode difference was more pronounced for mezzos than other categories (respectively +0.010, +0.134, +0.044, +0.133, +0.303, +0.149 for bass, baritone, tenor, alto, mezzo, soprano), resulting in an interaction between mode and category [*p*<*0.001*]. Gender did not result in a main effect [*p* = *0.766*] but did interact with voice category [*p*<*0.001*] as the TRANS group tended again to use a larger range of ratings than the CIS group. No other main effect or interaction was significant.

#### 4.2.3 AV-ratings

AV ratings were systematically shifted from A ratings, in the direction of V ratings. Once again, this is not obvious in Figure 2 or Figure 3 because the upward shifts are counteracted by the downward shifts but once signed, they were significantly above 0 [*t(236)* > *3.0, p*<*0.001 in all cases*] as depicted on the bottom panels of Figure 4. In other words, participants were *systematically biased in their appraisal of voices by the actors' physical appearance and gestures*. As a novel finding of the study: actors manipulated with longer VTL suffered more bias than (the same) actors with shorter VTL [*p*<*0.001*]. Marginal estimates were 0.284 [0.253–0.315] for shorter VTL, and 0.351 [0.320–0.382] for longer VTL. The VTL effect was complex because it affected actors with variable strength [*p*<*0.001*] typically affecting basses/baritones/tenors more than altos/mezzos/sopranos, and it was more accentuated when actors sang (0.360 vs. 0.247) than when they spoke (0.342 vs. 0.320) [*p*<*0.001*], in line with the observation made in Experiment 1 (bottom-right vs. bottom-left, Figure 4). Finally, the three factors interacted [*p* = *0.008*].

Importantly, TRANS individuals were less prone to the visual bias compared to CIS individuals [*p* = *0.004*] replicating our key earlier observation (Marchand Knight et al., 2023) and this trans advantage was larger for some actors than others [*p* = *0.004*]. The group difference was significant for basses [*p*<*0.001*], altos [*p*<*0.001*], mezzos [*p* = *0.005*], and sopranos [*p* = *0.025*], but it was not significant for baritones [*p* = *0.727*] and tenors [*p* = *0.236*]. Gender did not interact with any other factor or combination of factors [*p* > *0.125*].

### 4.3 Relationship to gender views

**Figure 5:**
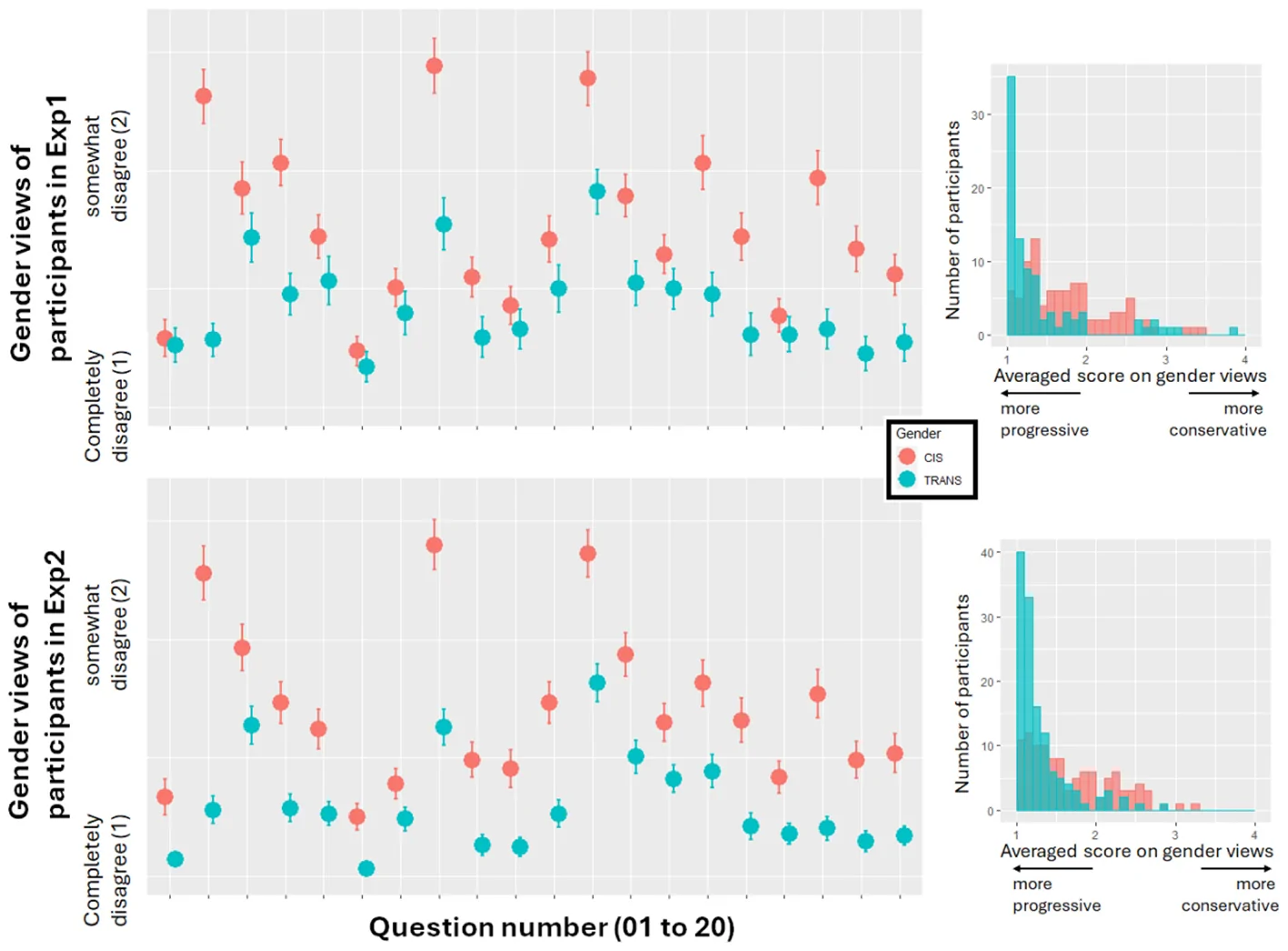
Scores obtained by cis (red) and trans (blue) participants in the questionnaire evaluating gender views, with means and standard errors in each population, for experiment 1 (**top-left** panel) and experiment 2 (**bottom-left** panel). Histograms (**right** panels) of the averaged score across the 20 questions in each population.

Figure 5 shows the responses to the questionnaire on gender views. In the first experiment, there was a main effect of gender [χ^2^(1)=14.0, p < 0.001], a main effect of questions [χ^2^(19) = 1011.8; *p* < 0.001], and a significant interaction [χ^2^(19) = 236.1, *p* < 0.001]. The effect of gender was significant in 11 out of the 20 questions [*p* < 0.025], but not significant for questions 01, 03, 05, 06, 07, 10, 11, 14, 17 [*p* > 0.084]. In the second experiment, there was a main effect of gender [χ^2^(1) = 47.5, *p* < 0.001], a main effect of questions [χ^2^(19) = 1,308.2, *p* < 0.001], and a significant interaction [χ^2^(19) = 117.7, *p* < 0.001]. The effect of gender was significant in 19 out of the 20 questions [*p* < 0.009], but not in question 07 [*p* = 0.107]. To summarize, results were qualitatively similar in both experiments (and similar to our previous study): TRANS participants held more progressive views on gender than CIS participants, and this was more apparent in some questions than in others.

**Figure 6:**
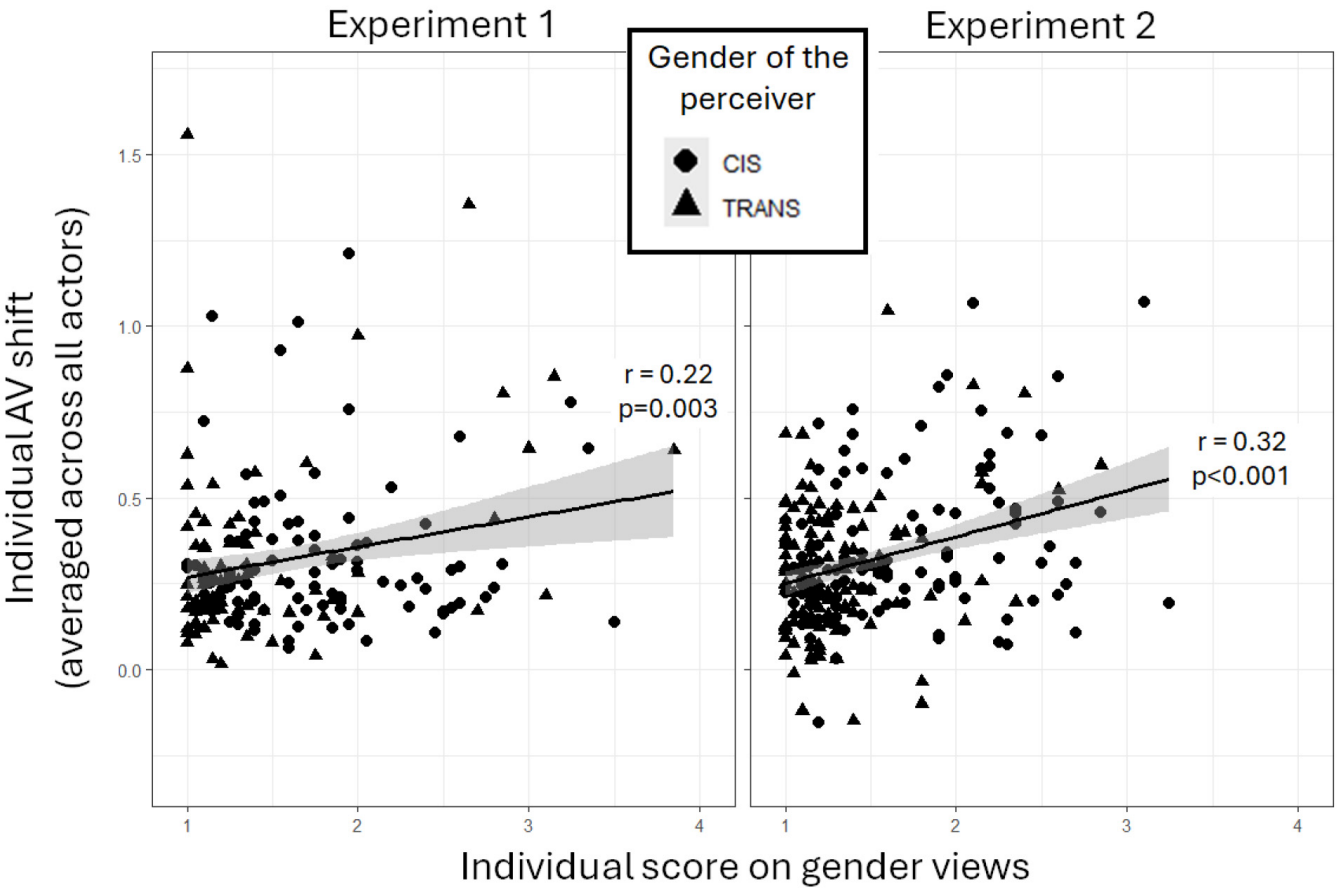
Correlation between individuals' score on gender views and their average AV shift, representing the degree to which a given participant was biased in their voice appraisal by the actors' physical appearance.

Linear regressions were conducted between the AV shift of each participant (averaged across all actors and mode) with their score on gender views. Figure 6 illustrates consistent links in the two experiments (surviving Bonferroni adjustments): when a participant held a greater binary view on gender and believed more strongly in traditional gender roles, they were more biased by visual cues in their appraisal of AV voices. This came a bit as a surprise because this association failed in our previous study, but it reinforced the idea that the phenomenon at play is related to cultural or societal views (which were perhaps broader in this study, having allowed for a broader age range for eligibility).

## 5 Discussion

In our previous paper, we established that people's impressions of voice type were influenced by visual cues but that this was less the case for TRANS than CIS individuals. Here we aimed to explore this visual bias phenomenon further and understand the conditions in which TRANS individuals appear more immune to it compared to CIS individuals. We did this by *manipulating* pitch and timbre aspects of the voice, independently, such that the visual cues would be precisely identical for lower/higher and darker/brighter voices. For audio-alone ratings, we found that both groups were equally influenced by pitch manipulations (suggesting a more universal cue) but that the TRANS group was more sensitive to timbre manipulations than the CIS group (suggesting a culturally ingrained cue). For video-alone ratings, we confirmed that the pitch/timbre manipulations had no effect. And for AV ratings, we replicated strong biases induced by the physical appearance of actors, but these biases occurred to different degrees in different participants, different degrees across actors, and different degrees whether actors spoke or sang. Specifically, we confirmed a trans advantage in Experiment 2 but not in Experiment 1. Thus, the current findings refine our previous interpretation.

### 5.1 Relative roles of pitch and timbre in voice appraisal: are they similarly effective?

The VTL manipulation had almost twice the effect of the F0 manipulation, namely +1.038 steps on the 6-point scale vs. +0.552 step (left panels of Figure 3). On this basis, we could say that VTL cues are more effective than F0 cues in influencing evaluation of voices. This is also consistent with the fact that vocal timbre alone can be used for gender categorization but not pitch alone, making timbre indeed a stronger gender marker (Roos et al., 2021; Pernet and Belin, 2012; Rendall et al., 2005; Titze, 1994). It follows that trans individuals would make a judicious choice in focussing on voice timbre to present themselves as intended.

But note that leveraging these vocal cues for an intended purpose is not unique to trans individuals. Pemberton et al. (1998) reported that women's habitual speaking pitch had lowered from 318 Hz (approximately E4) in 1924 to 206 Hz (approximately G3) by 1993. While this information is outdated and deserves further investigation, Watson (2019) suggested that women are seeking the help of speech language pathologists to sound more authoritative in the workplace and that the tactics being employed are both to lower pitch and “deepen” or “round out” the timbre, especially if lowering pitch is deemed impossible or unhealthy for the voice. This further supports the idea that individuals modulate pitch or timbre somewhat interchangeably, often for an intended purpose. In reconsidering the effects of helium and sulfur hexafluoride on the voice, much of the gray literature refers to helium making the voice higher and sulfur hexafluoride making the voice lower. These are, put simply, misnomers. The speaker's accessible pitch range remains roughly the same, but the listener's perception is modified by the change in timbre, produced by a change in reinforced harmonics and sound traveling through lighter (in the case of helium) or heavier (in the case of sulfur hexafluoride) gas. The fact that listeners confuse timbre with pitch can be used to our benefit in training trans voice users who wish to align voice and gender presentation. By brightening (reinforcing upper harmonics) or darkening (reinforcing lower harmonics) timbre, speakers can project a seemingly higher or lower voice even while the speaking F0 does not change.

### 5.2 Male voices subject to more prejudice than female voices

There is a sense in our data that male voices are generally subject to more prejudice than female voices. Somehow, the characteristics of a low and dark voice must set up expectations about excessively masculine physical traits in the face or body of the actor. When participants saw the actual male actors in action (speaking or singing), they no longer assigned their voice as much masculinity as it received in the audio-alone version. We had already observed this phenomenon across the 18 actors and discussed it (section 4.5 in Marchand Knight et al., 2023). The strength of the current study was to demonstrate it *within the same actors*. Here, in experiment 2, actors with longer VTL suffered more prejudice than *the same actors* with shorter VTL. Furthermore, this was particularly true for basses, baritones, and tenors. Comparatively, it must be easier for female voices to be in adequation with the participants' expectation of their physical appearance. Perhaps, this is because feminine boys and men are more punished in society than masculine girls and women. For example, boys who play with dolls are typically subject to greater prejudice than girls who excel at sports. Typically, when gender neutral attire is suggested, everyone wears pants (not skirts). Strauss (2018) describes gender progress as “a one-way street” where girls are allowed to engage with masculine things, but boys are still discouraged from engaging with things considered feminine (Savin-Williams, 2016). This phenomenon, called androcentrism, is the devaluing of feminine attributes. While all genders suffer due to androcentrism, they suffer differently. Selin Davis (2018) says it adequately:

“The answer is that we have internalized a kind of sexism that values masculinity in both boys and girls, just as it devalues femininity in them.”

Therefore, in our experiments, when participants heard what they presumed to be masculine voices, they could have created a mental image of an actor with a hyper-masculine appearance, and they could have reacted negatively once confronted with the actual physical appearance that transgressed their expectations. We believe that this is the root cause of these observations which strengthens the collective and cultural world that exists within voice perception, beyond traditional psychoacoustics research.

Moreover, this masculinity bias was more pronounced when actors sang. Indeed, in experiment 1, singing voices with lower F0 (more masculine-perceived) suffered more bias against them than singing voices with higher F0 (more feminine-perceived); and in experiment 2, singing voices with longer VTL (more masculine-perceived) suffered more bias against them than singing voices with shorter VTL (more feminine-perceived). It is worth questioning why this phenomenon is exacerbated in singing. One consistent finding observed here, in both experiments (as well as in our previous study), is the effect of mode: sung stimuli are perceived as higher/brighter. While this might seem somewhat intuitive for audio-alone stimuli, it was still the case (to a smaller degree) with silent videos. We speculate that actors are more expressive (auditorily or visually) when singing, and emotional expressivity tends to be associated with feminine traits (Hardy et al., 2020; Jackson Hearns and Kremer, 2018; Chaplin, 2015). Typically, the study by (Behne and Wöllner 2011)—where women pianists were perceived to play more expressively than men pianists (although the audio content was identical)—falls in line with the current observation. Notably, this association (singing-to-feminine) was slightly exacerbated by CIS viewers compared to TRANS viewers. Due to this association, it is harder for male actors' voice to be in adequation with participants' expectation of their physical appearance. So, this masculinity bias gets amplified in singing. Also, remember that the sung melodic pattern was fixed for all actors, with an *octave difference* between the three bottom (traditionally male) and three top (traditionally female) categories. So, F0 cues in the sung stimuli could have elicited a more binary division between basses/baritones/tenors on one side and altos/mezzos/sopranos on the other. As a result, the expectation of voices was perhaps even more gendered than in ecological contexts when actors spoke normally (with F0 variability creating a degree of crossing between male and female clusters).

This is very interesting as it resonates with the idea that a musical environment (e.g., auditioning for a role in opera) places the exact conditions for voice expectations to be even more gendered than needed. This creates unrealistic expectations about what a bass actor should look like, something that is less obvious outside of a musical environment. While opera is a medium that can be thought of as pushing gender boundaries, it does so only according to very strict conventions. Currently women mezzo-sopranos often play trouser roles (characters that are teenage boys at sexual awakening like Cherubino in Mozart's *Le nozze di Figaro* and Oktavian in Strauss's *Der Rosekavalier*) (both operas that JMK has performed and studied) and more heroic roles previously sung by castrati, men castrated in boyhood to preserve the treble singing voice, (such as the title role in Handel's *Giulio Cesare*, a role that JMK has performed). Skirt roles for men hardly exist today but sometimes a tenor may play the part of the Witch in Humperdinck's *Hansel and Gretel* or a countertenor may play the role of the bearded woman Baba the Turk in Stravinski's *The Rake's Progress*. These are usually reserved for comedic effect. Countertenor itself is an ambiguous term usually used to describe cis men who sing in the treble range—without any of the nuance applied to other *Fach* categories (coloratura, lyric, or dramatic, for example) and in fact, the term does not even account for the differences between countertenors who sing in the mezzo-soprano range or the alto range. Recently, the term sopranist has begun to appear to describe cis men singing in the soprano range, again without any nuance regarding vocal agility or weight. The terms countertenor and sopranist, in fact, point to a societal desire to categorize voices purely along gendered lines.

### 5.3 Trans advantage only partly replicated

We now turn to the group differences with a major question: why did we replicate the trans advantage in Experiment 2 but not in Experiment 1? First, this study was conducted online exclusively (like our previous study), so we never met or discussed gender identity with any of the participants. Unfortunately, we cannot exclude the possibility that some participants lied about their gender identities. We noticed some inconsistencies between their Prolific data (which included a gender selection) and what they reported during the study (as gender identity was directly asked in the online protocol). Such inconsistencies, however, are not necessarily evidence of fraud. Trans and gender fluid individuals may have come out after entering their gender in their Prolific profiles and for some, gender identity is fluid throughout life (hence the data gathered from their Prolific profile might have been outdated). This is hardly evidence of deception, so we relied foremost on the participants' direct input about gender identity. That is, we do not think the trans advantage disappeared in Experiment 1 due to falsified gender identity information.

Secondly, the set of stimuli used here was twice smaller than in our previous study. Half of the materials had to be removed in order to duplicate each stimulus with high vs. low F0, and short vs. long VTL and to do so, we selected only the emotionally neutral stimuli. So, we might suspect that the stimuli with higher emotional valence elicited a stronger trans advantage, but we had assessed this possibility in our previous study without much support for it.

A third explanation for this partial replication may be that the trans advantage is somewhat lost with artificial or manipulated stimuli. While a 3-semitones manipulation in F0 or VTL remained “believable,” it did make certain voices sound rather child-like and others large or menacing. This is a consequence of controlling this phenomenon in the lab; it is not trivial to do so while maintaining naturalness of ecological stimuli. Participants were not asked to rate the naturalness of the voices, and we assumed, here, that certain stimuli which sounded exaggerated by the manipulation could have forced a break with AV integration. Furthermore, different individuals have different auditory/musical abilities: some might have very fine difference limen (DL) to F0 and others fine DL to VTL. No attempt was made here to equate the manipulations to each participant's sensitivity to F0 or VTL. The manipulation could have been very salient in one participant, and much less in another. In fact, we have support for this idea here since we found that the F0 manipulations had a similar impact on both groups in Audio-alone ratings, but the VTL manipulations had more impact on trans than cis listeners. This implies that timbre cues might be perceptually more salient to trans individuals. A more salient cue is easier to rely on, allowing to better ignore the actors' physical appearance. So, this seems a likely explanation as to why we observed the trans advantage in Experiment 2 but not Experiment 1. This is not to say that trans people have an innate desire or ability to “transvestigate” or figure out gender (Serano, 2024) or even that they are looking harder than cis people for gender clues, but rather that they have been exposed to gender variances outside of the rigid binary that perhaps cis individuals have not (Iantaffi and Bockting, 2011). As Serano points out, perceiving most individuals as cisgender or of a strictly binary gender says more about the perceiver's life experience and knowledge than anything about the perceived person's actual identity (2024). If cisgender people have never been exposed to gender expansive people, they would naturally categorize according to the cues and categories they know.

To summarize, the lack of replication of the trans advantage in experiment 1 could be a combination of (1) less ecological and more neutral stimuli, and (2) the fact that pitch cues have a more universal impact on voice appraisal.

### 5.4 Considerations on age

In this study, we broadened our eligibility criterion to adults up to 70 years of age, in the hope that we would enhance the generalization of findings and inclusiveness. Unfortunately, it was not possible for Prolific to recruit enough older individuals within our TRANS batches. As a result, the two groups were slightly mismatched in age (4–5 years difference in mean age). We do not think this is a major flaw, yet this limitation deserves some consideration given that we failed to replicate the trans advantage in Experiment 1. Here, we discuss how age may have interacted with the present factors.

Just as older lesbian, gay, and straight people alike had a difficult time adjusting to the notion that sexual orientation exists on a spectrum rather than in a binary realm (Stewart, 2021; Pennasilico, 2019), it can be argued that older gender expansive and older cis individuals have a difficult time adjusting to gender existing on a spectrum rather than a binary. Gender identities such as non-binary, agender, gender fluid, and flux fluid were not articulated or defined for older Millennials, Gen X, or Baby Boomers while they were growing up. Younger Millennials, Gen Z, and Gen Alpha, on the other hand, are more aware of the notion of gender as fluid and gendered behaviors as constructed or imposed. While older trans individuals are certainly accepting of binary transitude, they may not fully understand non-binary identities because there was no language surrounding this identity when they were growing up. The restrictive views on gender in the 1950s certainly did not help. In order to transition, one had to pick man or woman; and one had to perform man or woman thoroughly as a matter of personal safety. Today, gender-affirming voice teachers acknowledge that signaling *cis* femininity, for example, is not always the trans feminine client's goal. Trans culture is not necessarily the same as cis culture anymore, and this has made it possible for trans people to choose which parts of their gender expression they wish to modify. It has made space for non-binary individuals to live in the center of the spectrum. However, this also created a certain level of tension between the trans philosophy of Baby Boomers and that of Gen Z. On this basis, it is possible that our current TRANS samples were substantially more heterogeneous than in our previous study, and therefore could have contributed to the lack of replication in Experiment 1. This being said, these limitations would apply just as much to Experiment 2, and yet the trans advantage was observed there. So, this limitation does not really jeopardize the emphasis of timbre cues over pitch cues as the root of the trans advantage.

### 5.5 Substantiation of voice timbre research

As the trans advantage points to a better use of timbre cues in voice, we feel inclined to say a few more words on research in this field, which is rather scant. As Yarnall (2017) says,

“*As it happens, little research has been conducted using empirical methods to explore vocal timbre*,” (2018).

Cleveland (1977) described voice timbre as, “That particular attribute of a given voice, which distinguishes that voice from another, when the vowel and the pitch are the same,” and established that tenor voices had higher formant frequencies than basses and baritones. Sundberg (1977) offered that gender differentiation in voice is due to women having a “smoother” timbre and men having a “rougher” timbre and that women speak approximately one octave higher than men. Timbre semantics draw largely on cross-modal metaphor, such as tactile qualities, colors, and temperatures. Mainka et al. (2015) did more work on vowels and vocal tract position as creators of timbre, but similarly to Cleveland, with only tenor and baritone participants (2015). Roos et al. (2021) point to timbre as the more significant gender marker (2021) and our work substantiates that timbre can be used as sophisticated way to signal gender.

Information about habitual speaking pitch, however, has changed significantly since the 1950s, with women now speaking lower (Pemberton et al., 1998). Changes in habitual speaking pitch, timbre, and even word choice has been attributed to women entering the workforce and adopting more assertive (read, masculine) manners of speech (Watson, 2019). An area of study that has made strong contributions to singing voice timbre research is popular music studies. Kate Heidemann posits that we might begin to describe vocal timbre through the embodied practice of mimesis. She raises important issues of individual perception and a lack of shared, agreed upon timbre semantics. Barna and McLaughlin (2024) echo Heidemann's approach while also turning to Malawey's timbre semantics (Malawey, 2020), as well as non-gendered timbre semantics terminology suggested by Marchand Knight and Marchand (2020). Reymore et al. (2026) confirmed that trained singers tend toward an embodied description of vocal timbre, often mentioning elements of vocal production and technique while attempting to describe timbre. This is something instrumentalists did less often, instead sticking to more common timbre semantics such as bright/dark. Noble (2022) demonstrated effectively, through spectral analysis, that one singer (JMK) can produce many timbres (breathy/folk; operatic/bel canto; music theater/belt) on a single pitch (A440 Hz), which is promising for gender-affirming voice teachers. Meanwhile, Ambros et al. (2025) have contributed work that incorporates room acoustics into vocal timbre analysis and vocal blending. Rossing and Sundberg (1984) and Skelton (2004) are concerned with this timbral notion of blend and the differences in vocal production for solo and ensemble singing, which again points to our ability to vary our timbre at will. Much of this has to do with the use or suppression of vibrato (Nestorova et al., in press). Still much of what is written about voice timbre is political or philosophical (rather than empirical) in nature, investigating the relationship of perceived race, ethnicity, gender, and sexual orientation to voice timbre (Marchand Knight and Marchand, 2020; Eidsheim, 2019; Barthes, 2009; Olwage, 2004). Our work here seeks to add to the growing body of empirical research on timbre, and more specifically, vocal timbre.

## 6 Conclusion

When explicitly asking participants to judge a voice, they cannot help but be influenced by how the actor looks. This visual bias is unfortunate but is presumably an inherent facet of the audio-visual integration capabilities of the brain. Interestingly, not everyone is subject to this bias to the same degree: some are greatly influenced while others are less prone to it. Our previous study suggested that trans individuals are likely to belong to the latter group. This study managed to confirm it in experiment 2 but not in experiment 1. The most likely explanation for this partial replication of the trans advantage is that timbre cues are more salient to trans individuals than to cis individuals (seen in Audio-alone appraisal) while pitch cues are equally salient to both groups. As such, it is conceivable that trans individuals downregulate the influence of physical appearance by upregulating the role of voice timbre.

## Data Availability

The raw data supporting the conclusions of this article will be made available by the authors, without undue reservation.

## Footnotes

1 Although VTL is normally reported in inches or centimeters (i.e., a measure of length), here it was expressed in semitone units, by dividing a vocal tract twice longer into 12 logarithmic steps, just like for pitch.
